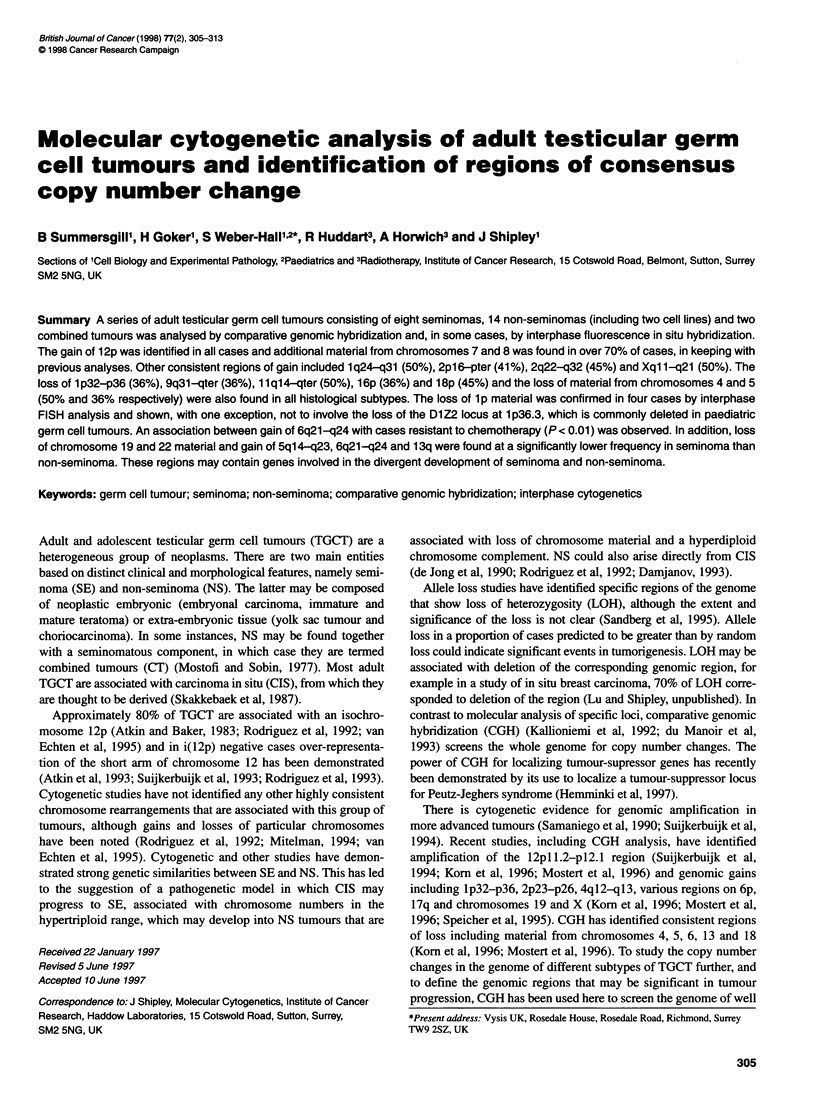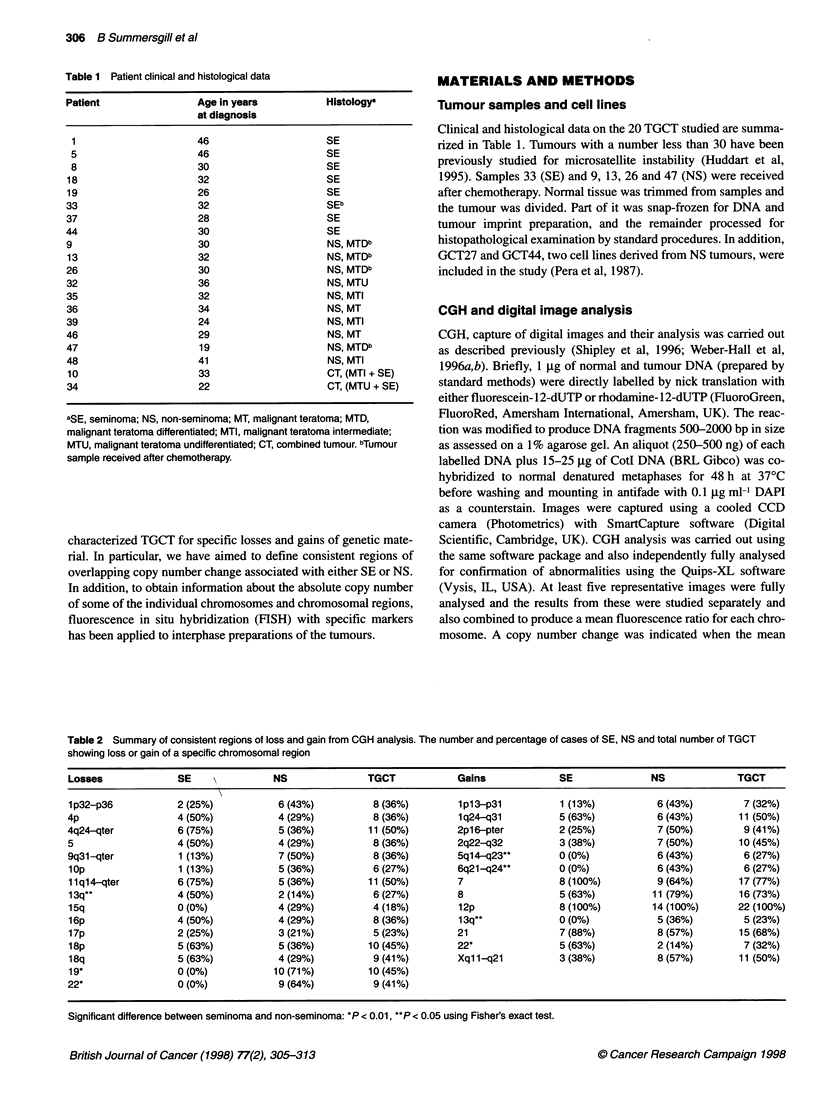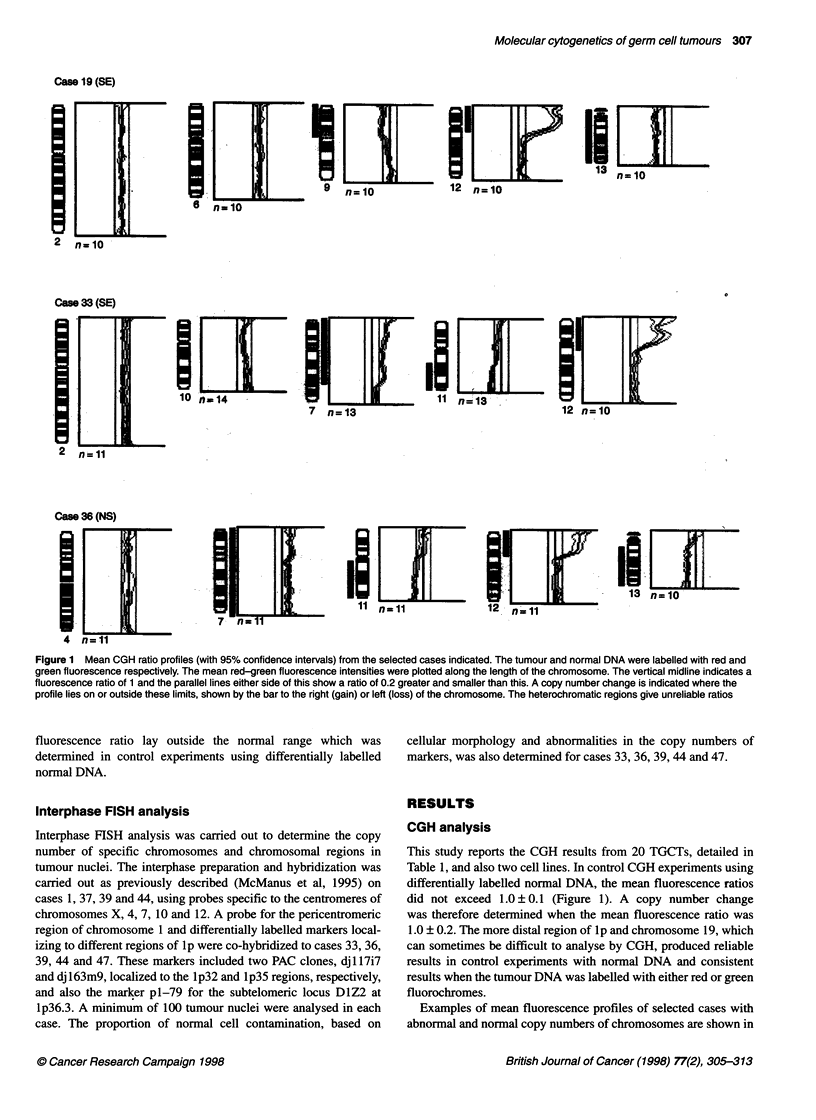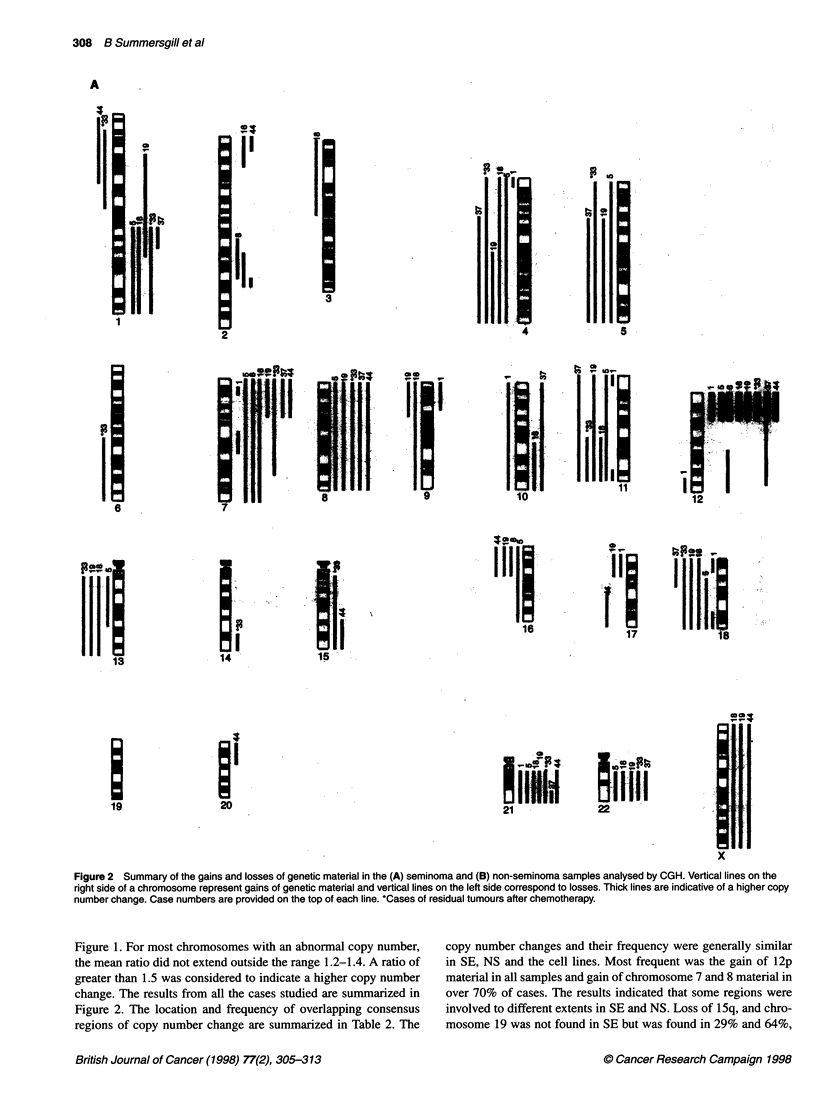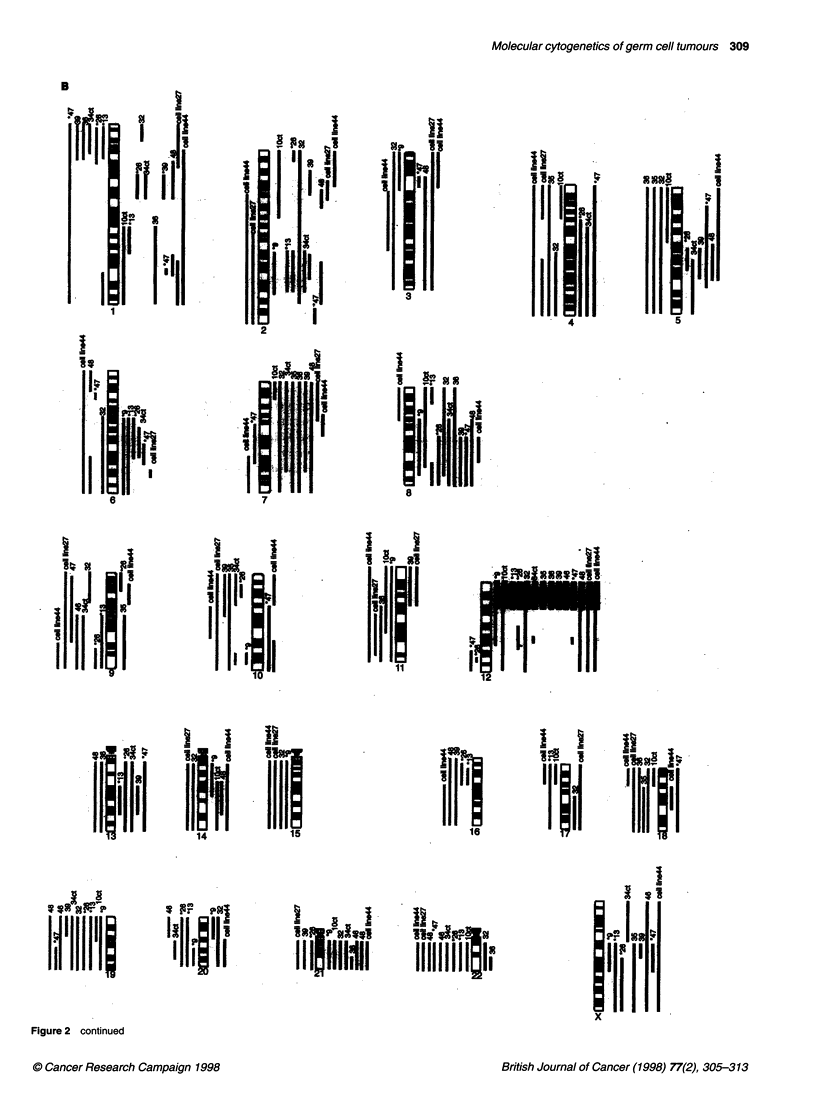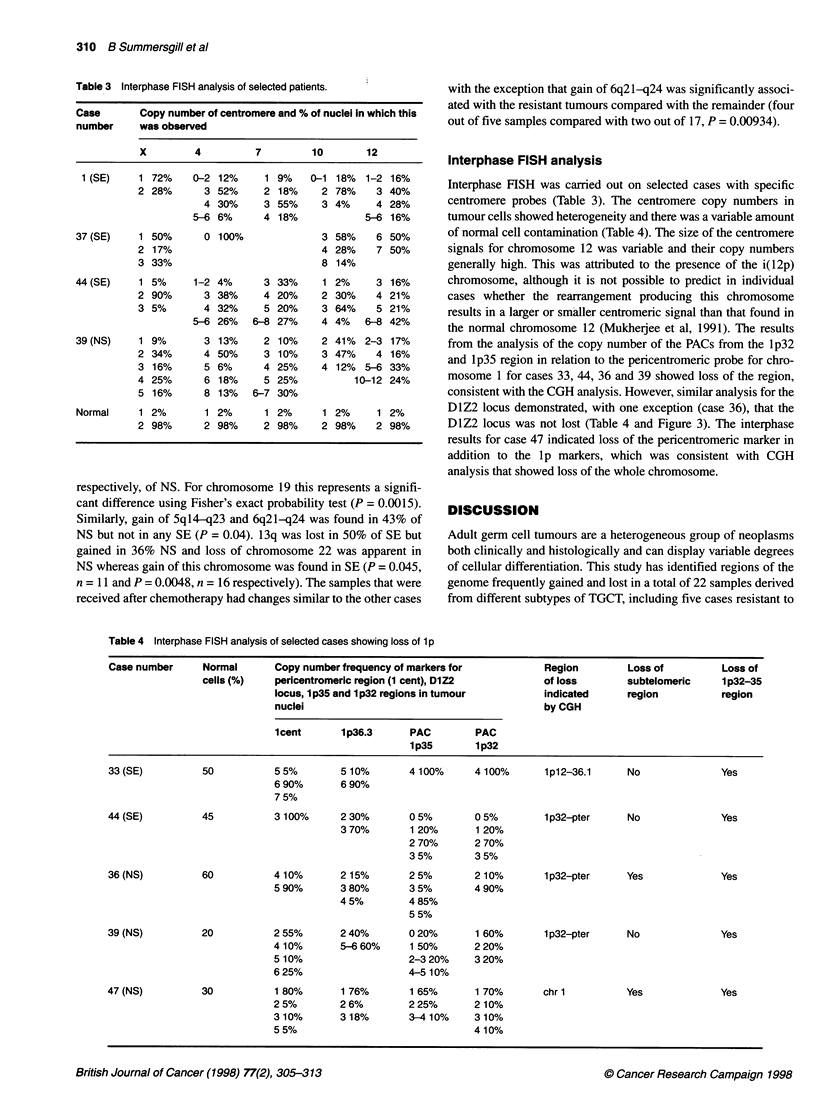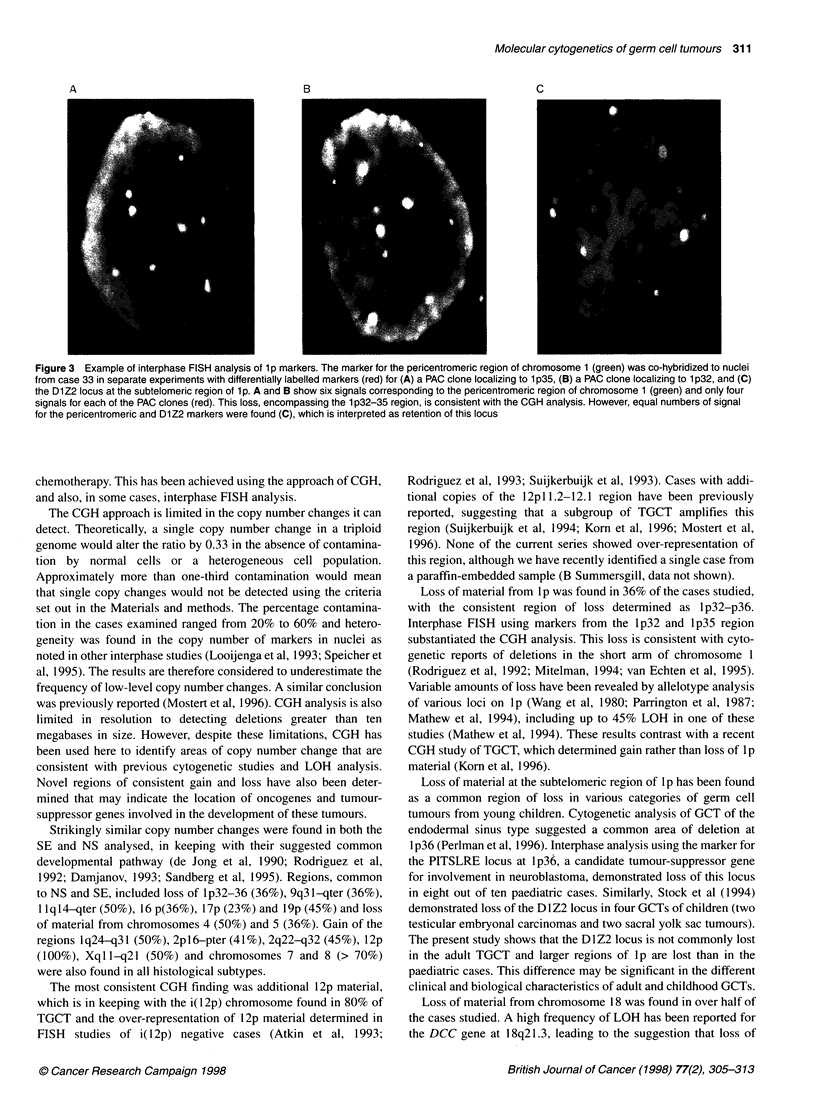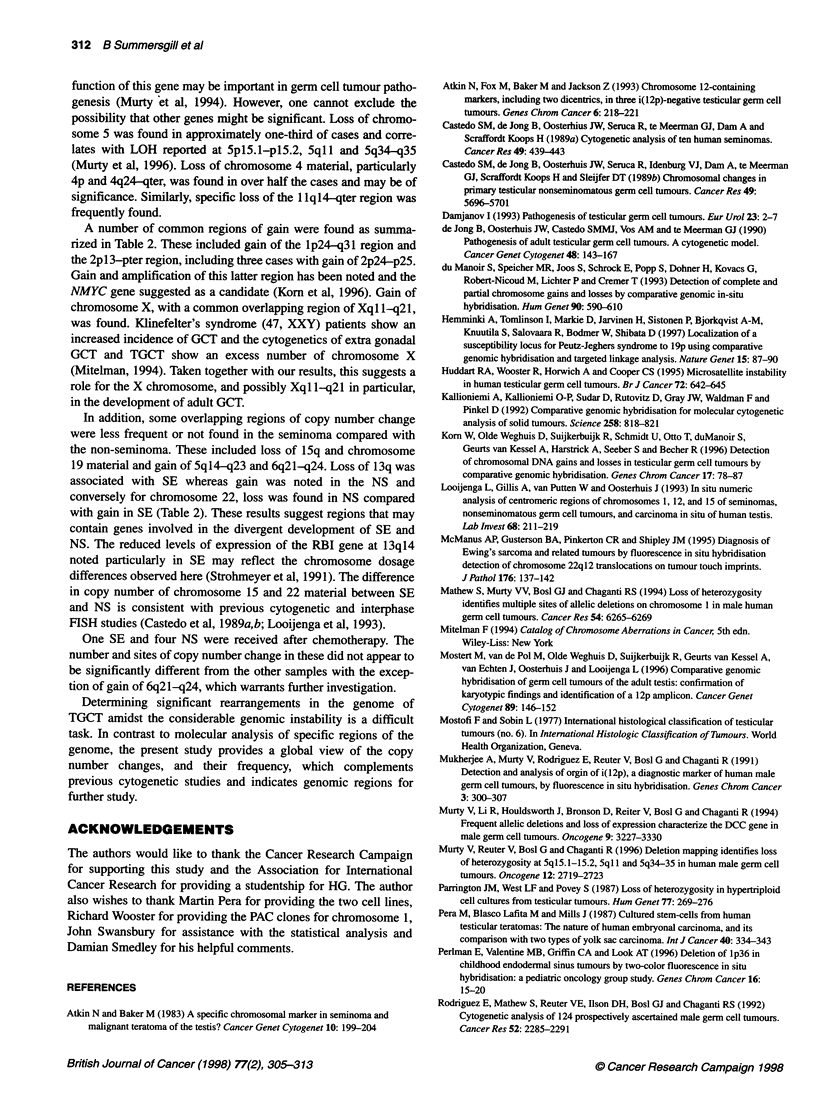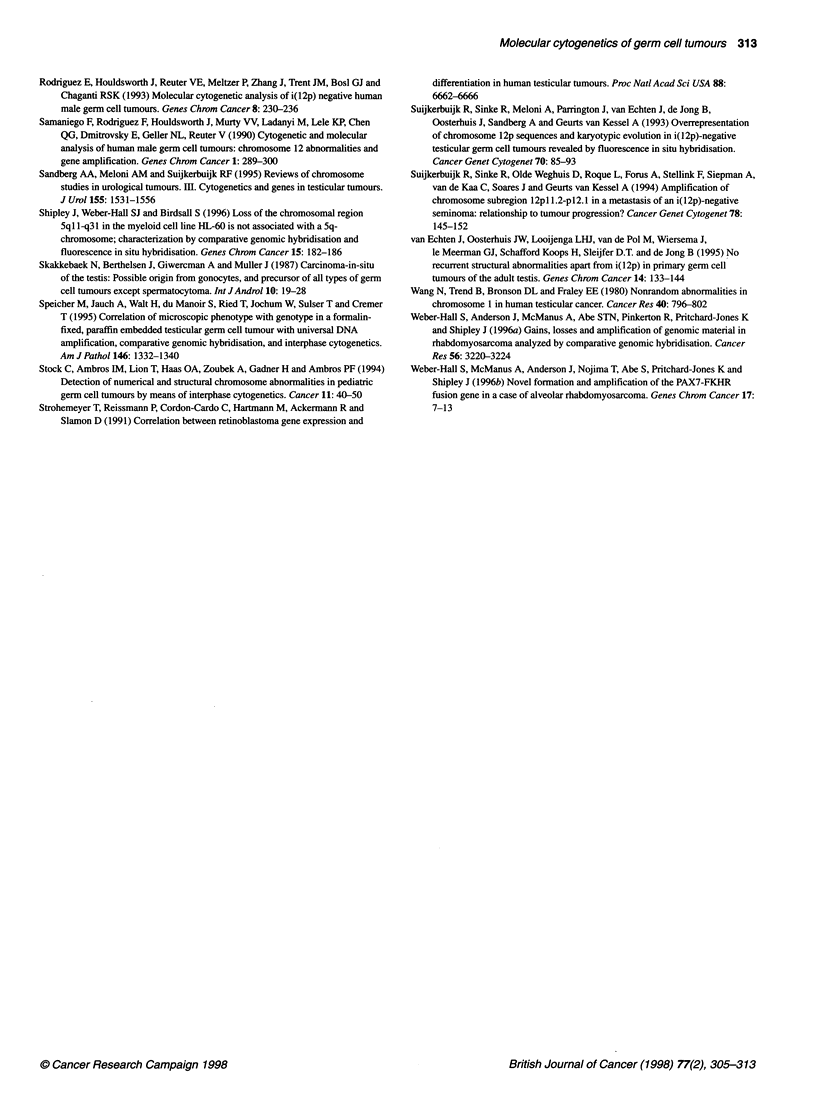# Molecular cytogenetic analysis of adult testicular germ cell tumours and identification of regions of consensus copy number change.

**DOI:** 10.1038/bjc.1998.47

**Published:** 1998

**Authors:** B. Summersgill, H. Goker, S. Weber-Hall, R. Huddart, A. Horwich, J. Shipley

**Affiliations:** Section of Cell Biology, Institute of Cancer Research, Sutton, Surrey, UK.

## Abstract

**Images:**


British Journal of Cancer (1998) 77(2), 305–313
© 1998 Cancer Research Campaign

### Molecular cytogenetic analysis of adult testicular germ cell tumours and identification of regions of consensus copy number change

**B Summersgill[1], H Goker[1], S Weber-Hall[1,2*], R Huddart[3], A Horwich[3] and J Shipley[1]**

Sections of [1]Cell Biology and Experimental Pathology, [2]Paediatrics and [3]Radiotherapy, Institute of Cancer Research, 15 Cotswold Road, Belmont, Sutton, Surrey SM2 5NG, UK

**Summary** A series of adult testicular germ cell tumours consisting of eight seminomas, 14 non-seminomas (including two cell lines) and two combined tumours was analysed by comparative genomic hybridization and, in some cases, by interphase fluorescence in situ hybridization. The gain of 12p was identified in all cases and additional material from chromosomes 7 and 8 was found in over 70% of cases, in keeping with previous analyses. Other consistent regions of gain included 1q24–q31 (50%), 2p16–pter (41%), 2q22–q32 (45%) and Xq11–q21 (50%). The loss of 1p32–p36 (36%), 9q31–qter (36%), 11q14–qter (50%), 16p (36%) and 18p (45%) and the loss of material from chromosomes 4 and 5 (50% and 36% respectively) were also found in all histological subtypes. The loss of 1p material was confirmed in four cases by interphase FISH analysis and shown, with one exception, not to involve the loss of the D1Z2 locus at 1p36.3, which is commonly deleted in paediatric germ cell tumours. An association between gain of 6q21–q24 with cases resistant to chemotherapy ($P < 0.01$) was observed. In addition, loss of chromosome 19 and 22 material and gain of 5q14–q23, 6q21–q24 and 13q were found at a significantly lower frequency in seminoma than non-seminoma. These regions may contain genes involved in the divergent development of seminoma and non-seminoma.

**Keywords:** germ cell tumour; seminoma; non-seminoma; comparative genomic hybridization; interphase cytogenetics

Adult and adolescent testicular germ cell tumours (TGCT) are a heterogeneous group of neoplasms. There are two main entities based on distinct clinical and morphological features, namely seminoma (SE) and non-seminoma (NS). The latter may be composed of neoplastic embryonic (embryonal carcinoma, immature and mature teratoma) or extra-embryonic tissue (yolk sac tumour and choriocarcinoma). In some instances, NS may be found together with a seminomatous component, in which case they are termed combined tumours (CT) (Mostofi and Sobin, 1977). Most adult TGCT are associated with carcinoma in situ (CIS), from which they are thought to be derived (Skakkebaek et al, 1987).

Approximately 80% of TGCT are associated with an isochromosome 12p (Atkin and Baker, 1983; Rodriguez et al, 1992; van Echten et al, 1995) and in i(12p) negative cases over-representation of the short arm of chromosome 12 has been demonstrated (Atkin et al, 1993; Suijkerbuijk et al, 1993; Rodriguez et al, 1993). Cytogenetic studies have not identified any other highly consistent chromosome rearrangements that are associated with this group of tumours, although gains and losses of particular chromosomes have been noted (Rodriguez et al, 1992; Mitelman, 1994; van Echten et al, 1995). Cytogenetic and other studies have demonstrated strong genetic similarities between SE and NS. This has led to the suggestion of a pathogenetic model in which CIS may progress to SE, associated with chromosome numbers in the hypertriploid range, which may develop into NS tumours that are associated with loss of chromosome material and a hyperdiploid chromosome complement. NS could also arise directly from CIS (de Jong et al, 1990; Rodriguez et al, 1992; Damjanov, 1993).

Allele loss studies have identified specific regions of the genome that show loss of heterozygosity (LOH), although the extent and significance of the loss is not clear (Sandberg et al, 1995). Allele loss in a proportion of cases predicted to be greater than by random loss could indicate significant events in tumorigenesis. LOH may be associated with deletion of the corresponding genomic region, for example in a study of in situ breast carcinoma, 70% of LOH corresponded to deletion of the region (Lu and Shipley, unpublished). In contrast to molecular analysis of specific loci, comparative genomic hybridization (CGH) (Kallioniemi et al, 1992; du Manoir et al, 1993) screens the whole genome for copy number changes. The power of CGH for localizing tumour-supressor genes has recently been demonstrated by its use to localize a tumour-suppressor locus for Peutz-Jeghers syndrome (Hemminki et al, 1997).

There is cytogenetic evidence for genomic amplification in more advanced tumours (Samaniego et al, 1990; Suijkerbuijk et al, 1994). Recent studies, including CGH analysis, have identified amplification of the 12p11.2–p12.1 region (Suijkerbuijk et al, 1994; Korn et al, 1996; Mostert et al, 1996) and genomic gains including 1p32–p36, 2p23–p26, 4q12–q13, various regions on 6p, 17q and chromosomes 19 and X (Korn et al, 1996; Mostert et al, 1996; Speicher et al, 1995). CGH has identified consistent regions of loss including material from chromosomes 4, 5, 6, 13 and 18 (Korn et al, 1996; Mostert et al, 1996). To study the copy number changes in the genome of different subtypes of TGCT further, and to define the genomic regions that may be significant in tumour progression, CGH has been used here to screen the genome of well

*Received 22 January 1997*
*Revised 5 June 1997*
*Accepted 10 June 1997*

*Correspondence to:* J Shipley, Molecular Cytogenetics, Institute of Cancer Research, Haddow Laboratories, 15 Cotswold Road, Sutton, Surrey, SM2 5NG, UK

**Present address:* Vysis UK, Rosedale House, Rosedale Road, Richmond, Surrey TW9 2SZ, UK

**Table 1** Patient clinical and histological data

| Patient | Age in years at diagnosis | Histology[a] |
|---|---|---|
| 1 | 46 | SE |
| 5 | 46 | SE |
| 8 | 30 | SE |
| 18 | 32 | SE |
| 19 | 26 | SE |
| 33 | 32 | SE[b] |
| 37 | 28 | SE |
| 44 | 30 | SE |
| 9 | 30 | NS, MTD[b] |
| 13 | 32 | NS, MTD[b] |
| 26 | 30 | NS, MTD[b] |
| 32 | 36 | NS, MTU |
| 35 | 32 | NS, MTI |
| 36 | 34 | NS, MT |
| 39 | 24 | NS, MTI |
| 46 | 29 | NS, MT |
| 47 | 19 | NS, MTD[b] |
| 48 | 41 | NS, MTI |
| 10 | 33 | CT, (MTI + SE) |
| 34 | 22 | CT, (MTU + SE) |

[a]SE, seminoma; NS, non-seminoma; MT, malignant teratoma; MTD, malignant teratoma differentiated; MTI, malignant teratoma intermediate; MTU, malignant teratoma undifferentiated; CT, combined tumour. [b]Tumour sample received after chemotherapy.

characterized TGCT for specific losses and gains of genetic material. In particular, we have aimed to define consistent regions of overlapping copy number change associated with either SE or NS. In addition, to obtain information about the absolute copy number of some of the individual chromosomes and chromosomal regions, fluorescence in situ hybridization (FISH) with specific markers has been applied to interphase preparations of the tumours.

### MATERIALS AND METHODS

### Tumour samples and cell lines

Clinical and histological data on the 20 TGCT studied are summarized in Table 1. Tumours with a number less than 30 have been previously studied for microsatellite instability (Huddart et al, 1995). Samples 33 (SE) and 9, 13, 26 and 47 (NS) were received after chemotherapy. Normal tissue was trimmed from samples and the tumour was divided. Part of it was snap-frozen for DNA and tumour imprint preparation, and the remainder processed for histopathological examination by standard procedures. In addition, GCT27 and GCT44, two cell lines derived from NS tumours, were included in the study (Pera et al, 1987).

### CGH and digital image analysis

CGH, capture of digital images and their analysis was carried out as described previously (Shipley et al, 1996; Weber-Hall et al, 1996*a*,*b*). Briefly, 1 μg of normal and tumour DNA (prepared by standard methods) were directly labelled by nick translation with either fluorescein-12-dUTP or rhodamine-12-dUTP (FluoroGreen, FluoroRed, Amersham International, Amersham, UK). The reaction was modified to produce DNA fragments 500–2000 bp in size as assessed on a 1% agarose gel. An aliquot (250–500 ng) of each labelled DNA plus 15–25 μg of CotI DNA (BRL Gibco) was co-hybridized to normal denatured metaphases for 48 h at 37°C before washing and mounting in antifade with 0.1 μg $ml^{-1}$ DAPI as a counterstain. Images were captured using a cooled CCD camera (Photometrics) with SmartCapture software (Digital Scientific, Cambridge, UK). CGH analysis was carried out using the same software package and also independently fully analysed for confirmation of abnormalities using the Quips-XL software (Vysis, IL, USA). At least five representative images were fully analysed and the results from these were studied separately and also combined to produce a mean fluorescence ratio for each chromosome. A copy number change was indicated when the mean

**Table 2** Summary of consistent regions of loss and gain from CGH analysis. The number and percentage of cases of SE, NS and total number of TGCT showing loss or gain of a specific chromosomal region

| Losses | SE | NS | TGCT | Gains | SE | NS | TGCT |
|---|---|---|---|---|---|---|---|
| 1p32–p36 | 2 (25%) | 6 (43%) | 8 (36%) | 1p13–p31 | 1 (13%) | 6 (43%) | 7 (32%) |
| 4p | 4 (50%) | 4 (29%) | 8 (36%) | 1q24–q31 | 5 (63%) | 6 (43%) | 11 (50%) |
| 4q24–qter | 6 (75%) | 5 (36%) | 11 (50%) | 2p16–pter | 2 (25%) | 7 (50%) | 9 (41%) |
| 5 | 4 (50%) | 4 (29%) | 8 (36%) | 2q22–q32 | 3 (38%) | 7 (50%) | 10 (45%) |
| 9q31–qter | 1 (13%) | 7 (50%) | 8 (36%) | 5q14–q23** | 0 (0%) | 6 (43%) | 6 (27%) |
| 10p | 1 (13%) | 5 (36%) | 6 (27%) | 6q21–q24** | 0 (0%) | 6 (43%) | 6 (27%) |
| 11q14–qter | 6 (75%) | 5 (36%) | 11 (50%) | 7 | 8 (100%) | 9 (64%) | 17 (77%) |
| 13q** | 4 (50%) | 2 (14%) | 6 (27%) | 8 | 5 (63%) | 11 (79%) | 16 (73%) |
| 15q | 0 (0%) | 4 (29%) | 4 (18%) | 12p | 8 (100%) | 14 (100%) | 22 (100%) |
| 16p | 4 (50%) | 4 (29%) | 8 (36%) | 13q** | 0 (0%) | 5 (36%) | 5 (23%) |
| 17p | 2 (25%) | 3 (21%) | 5 (23%) | 21 | 7 (88%) | 8 (57%) | 15 (68%) |
| 18p | 5 (63%) | 5 (36%) | 10 (45%) | 22* | 5 (63%) | 2 (14%) | 7 (32%) |
| 18q | 5 (63%) | 4 (29%) | 9 (41%) | Xq11–q21 | 3 (38%) | 8 (57%) | 11 (50%) |
| 19* | 0 (0%) | 10 (71%) | 10 (45%) | | | | |
| 22* | 0 (0%) | 9 (64%) | 9 (41%) | | | | |

Significant difference between seminoma and non-seminoma: $^{*}P < 0.01$, $^{**}P < 0.05$ using Fisher's exact test.

© Cancer Research Campaign 1998

**Figure 1** Mean CGH ratio profiles (with 95% confidence intervals) from the selected cases indicated. The tumour and normal DNA were labelled with red and green fluorescence respectively. The mean red–green fluorescence intensities were plotted along the length of the chromosome. The vertical midline indicates a fluorescence ratio of 1 and the parallel lines either side of this show a ratio of 0.2 greater and smaller than this. A copy number change is indicated where the profile lies on or outside these limits, shown by the bar to the right (gain) or left (loss) of the chromosome. The heterochromatic regions give unreliable ratios fluorescence ratio lay outside the normal range which was determined in control experiments using differentially labelled normal DNA.

### Interphase FISH analysis

Interphase FISH analysis was carried out to determine the copy number of specific chromosomes and chromosomal regions in tumour nuclei. The interphase preparation and hybridization was carried out as previously described (McManus et al, 1995) on cases 1, 37, 39 and 44, using probes specific to the centromeres of chromosomes X, 4, 7, 10 and 12. A probe for the pericentromeric region of chromosome 1 and differentially labelled markers localizing to different regions of 1p were co-hybridized to cases 33, 36, 39, 44 and 47. These markers included two PAC clones, dj117i7 and dj163m9, localized to the 1p32 and 1p35 regions, respectively, and also the marker p1–79 for the subtelomeric locus D1Z2 at 1p36.3. A minimum of 100 tumour nuclei were analysed in each case. The proportion of normal cell contamination, based on cellular morphology and abnormalities in the copy numbers of markers, was also determined for cases 33, 36, 39, 44 and 47.

### RESULTS

### CGH analysis

This study reports the CGH results from 20 TGCTs, detailed in Table 1, and also two cell lines. In control CGH experiments using differentially labelled normal DNA, the mean fluorescence ratios did not exceed $1.0 \pm 0.1$ (Figure 1). A copy number change was therefore determined when the mean fluorescence ratio was $1.0 \pm 0.2$. The more distal region of 1p and chromosome 19, which can sometimes be difficult to analyse by CGH, produced reliable results in control experiments with normal DNA and consistent results when the tumour DNA was labelled with either red or green fluorochromes.

Examples of mean fluorescence profiles of selected cases with abnormal and normal copy numbers of chromosomes are shown in

© Cancer Research Campaign 1998

**Figure 2** Summary of the gains and losses of genetic material in the (**A**) seminoma and (**B**) non-seminoma samples analysed by CGH. Vertical lines on the right side of a chromosome represent gains of genetic material and vertical lines on the left side correspond to losses. Thick lines are indicative of a higher copy number change. Case numbers are provided on the top of each line. *Cases of residual tumours after chemotherapy.

Figure 1. For most chromosomes with an abnormal copy number, the mean ratio did not extend outside the range 1.2–1.4. A ratio of greater than 1.5 was considered to indicate a higher copy number change. The results from all the cases studied are summarized in Figure 2. The location and frequency of overlapping consensus regions of copy number change are summarized in Table 2. The copy number changes and their frequency were generally similar in SE, NS and the cell lines. Most frequent was the gain of 12p material in all samples and gain of chromosome 7 and 8 material in over 70% of cases. The results indicated that some regions were involved to different extents in SE and NS. Loss of 15q, and chromosome 19 was not found in SE but was found in 29% and 64%,

© Cancer Research Campaign 1998

**Figure 2** continued

**Table 3** Interphase FISH analysis of selected patients.

| Case number | Copy number of centromere and % of nuclei in which this was observed | | | | |
|---|---|---|---|---|---|
| | X | 4 | 7 | 10 | 12 |
| 1 (SE) | 1 72% | 0–2 12% | 1 9% | 0–1 18% | 1–2 16% |
| | 2 28% | 3 52% | 2 18% | 2 78% | 3 40% |
| | | 4 30% | 3 55% | 3 4% | 4 28% |
| | | 5–6 6% | 4 18% | | 5–6 16% |
| 37 (SE) | 1 50% | 0 100% | | 3 58% | 6 50% |
| | 2 17% | | | 4 28% | 7 50% |
| | 3 33% | | | 8 14% | |
| 44 (SE) | 1 5% | 1–2 4% | 3 33% | 1 2% | 3 16% |
| | 2 90% | 3 38% | 4 20% | 2 30% | 4 21% |
| | 3 5% | 4 32% | 5 20% | 3 64% | 5 21% |
| | | 5–6 26% | 6–8 27% | 4 4% | 6–8 42% |
| 39 (NS) | 1 9% | 3 13% | 2 10% | 2 41% | 2–3 17% |
| | 2 34% | 4 50% | 3 10% | 3 47% | 4 16% |
| | 3 16% | 5 6% | 4 25% | 4 12% | 5–6 33% |
| | 4 25% | 6 18% | 5 25% | | 10–12 24% |
| | 5 16% | 8 13% | 6–7 30% | | |
| Normal | 1 2% | 1 2% | 1 2% | 1 2% | 1 2% |
| | 2 98% | 2 98% | 2 98% | 2 98% | 2 98% |

respectively, of NS. For chromosome 19 this represents a significant difference using Fisher's exact probability test ($P = 0.0015$). Similarly, gain of 5q14–q23 and 6q21–q24 was found in 43% of NS but not in any SE ($P = 0.04$). 13q was lost in 50% of SE but gained in 36% NS and loss of chromosome 22 was apparent in NS whereas gain of this chromosome was found in SE ($P = 0.045$, $n = 11$ and $P = 0.0048$, $n = 16$ respectively). The samples that were received after chemotherapy had changes similar to the other cases with the exception that gain of 6q21–q24 was significantly associated with the resistant tumours compared with the remainder (four out of five samples compared with two out of 17, $P = 0.00934$).

### Interphase FISH analysis

Interphase FISH was carried out on selected cases with specific centromere probes (Table 3). The centromere copy numbers in tumour cells showed heterogeneity and there was a variable amount of normal cell contamination (Table 4). The size of the centromere signals for chromosome 12 was variable and their copy numbers generally high. This was attributed to the presence of the i(12p) chromosome, although it is not possible to predict in individual cases whether the rearrangement producing this chromosome results in a larger or smaller centromeric signal than that found in the normal chromosome 12 (Mukherjee et al, 1991). The results from the analysis of the copy number of the PACs from the 1p32 and 1p35 region in relation to the pericentromeric probe for chromosome 1 for cases 33, 44, 36 and 39 showed loss of the region, consistent with the CGH analysis. However, similar analysis for the D1Z2 locus demonstrated, with one exception (case 36), that the D1Z2 locus was not lost (Table 4 and Figure 3). The interphase results for case 47 indicated loss of the pericentromeric marker in addition to the 1p markers, which was consistent with CGH analysis that showed loss of the whole chromosome.

### DISCUSSION

Adult germ cell tumours are a heterogeneous group of neoplasms both clinically and histologically and can display variable degrees of cellular differentiation. This study has identified regions of the genome frequently gained and lost in a total of 22 samples derived from different subtypes of TGCT, including five cases resistant to

**Table 4** Interphase FISH analysis of selected cases showing loss of 1p

| Case number | Normal cells (%) | Copy number frequency of markers for pericentromeric region (1 cent), D1Z2 locus, 1p35 and 1p32 regions in tumour nuclei | | | | Region of loss indicated by CGH | Loss of subtelomeric region | Loss of 1p32–35 region |
|---|---|---|---|---|---|---|---|---|
| | | 1cent | 1p36.3 | PAC 1p35 | PAC 1p32 | | | |
| 33 (SE) | 50 | 5 5% | 5 10% | 4 100% | 4 100% | 1p12–36.1 | No | Yes |
| | | 6 90% | 6 90% | | | | | |
| | | 7 5% | | | | | | |
| 44 (SE) | 45 | 3 100% | 2 30% | 0 5% | 0 5% | 1p32–pter | No | Yes |
| | | | 3 70% | 1 20% | 1 20% | | | |
| | | | | 2 70% | 2 70% | | | |
| | | | | 3 5% | 3 5% | | | |
| 36 (NS) | 60 | 4 10% | 2 15% | 2 5% | 2 10% | 1p32–pter | Yes | Yes |
| | | 5 90% | 3 80% | 3 5% | 4 90% | | | |
| | | | 4 5% | 4 85% | | | | |
| | | | | 5 5% | | | | |
| 39 (NS) | 20 | 2 55% | 2 40% | 0 20% | 1 60% | 1p32–pter | No | Yes |
| | | 4 10% | 5–6 60% | 1 50% | 2 20% | | | |
| | | 5 10% | | 2–3 20% | 3 20% | | | |
| | | 6 25% | | 4–5 10% | | | | |
| 47 (NS) | 30 | 1 80% | 1 76% | 1 65% | 1 70% | chr 1 | Yes | Yes |
| | | 2 5% | 2 6% | 2 25% | 2 10% | | | |
| | | 3 10% | 3 18% | 3–4 10% | 3 10% | | | |
| | | 5 5% | | | 4 10% | | | |

© Cancer Research Campaign 1998

**Figure 3** Example of interphase FISH analysis of 1p markers. The marker for the pericentromeric region of chromosome 1 (green) was co-hybridized to nuclei from case 33 in separate experiments with differentially labelled markers (red) for (**A**) a PAC clone localizing to 1p35, (**B**) a PAC clone localizing to 1p32, and (**C**) the D1Z2 locus at the subtelomeric region of 1p. **A** and **B** show six signals corresponding to the pericentromeric region of chromosome 1 (green) and only four signals for each of the PAC clones (red). This loss, encompassing the 1p32–35 region, is consistent with the CGH analysis. However, equal numbers of signal for the pericentromeric and D1Z2 markers were found (**C**), which is interpreted as retention of this locus chemotherapy. This has been achieved using the approach of CGH, and also, in some cases, interphase FISH analysis.

The CGH approach is limited in the copy number changes it can detect. Theoretically, a single copy number change in a triploid genome would alter the ratio by 0.33 in the absence of contamination by normal cells or a heterogeneous cell population. Approximately more than one-third contamination would mean that single copy changes would not be detected using the criteria set out in the Materials and methods. The percentage contamination in the cases examined ranged from 20% to 60% and heterogeneity was found in the copy number of markers in nuclei as noted in other interphase studies (Looijenga et al, 1993; Speicher et al, 1995). The results are therefore considered to underestimate the frequency of low-level copy number changes. A similar conclusion was previously reported (Mostert et al, 1996). CGH analysis is also limited in resolution to detecting deletions greater than ten megabases in size. However, despite these limitations, CGH has been used here to identify areas of copy number change that are consistent with previous cytogenetic studies and LOH analysis. Novel regions of consistent gain and loss have also been determined that may indicate the location of oncogenes and tumour-suppressor genes involved in the development of these tumours.

Strikingly similar copy number changes were found in both the SE and NS analysed, in keeping with their suggested common developmental pathway (de Jong et al, 1990; Rodriguez et al, 1992; Damjanov, 1993; Sandberg et al, 1995). Regions, common to NS and SE, included loss of 1p32–36 (36%), 9q31–qter (36%), 11q14–qter (50%), 16 p(36%), 17p (23%) and 19p (45%) and loss of material from chromosomes 4 (50%) and 5 (36%). Gain of the regions 1q24–q31 (50%), 2p16–pter (41%), 2q22–q32 (45%), 12p (100%), Xq11–q21 (50%) and chromosomes 7 and 8 (> 70%) were also found in all histological subtypes.

The most consistent CGH finding was additional 12p material, which is in keeping with the i(12p) chromosome found in 80% of TGCT and the over-representation of 12p material determined in FISH studies of i(12p) negative cases (Atkin et al, 1993; Rodriguez et al, 1993; Suijkerbuijk et al, 1993). Cases with additional copies of the 12p11.2–12.1 region have been previously reported, suggesting that a subgroup of TGCT amplifies this region (Suijkerbuijk et al, 1994; Korn et al, 1996; Mostert et al, 1996). None of the current series showed over-representation of this region, although we have recently identified a single case from a paraffin-embedded sample (B Summersgill, data not shown).

Loss of material from 1p was found in 36% of the cases studied, with the consistent region of loss determined as 1p32–p36. Interphase FISH using markers from the 1p32 and 1p35 region substantiated the CGH analysis. This loss is consistent with cytogenetic reports of deletions in the short arm of chromosome 1 (Rodriguez et al, 1992; Mitelman, 1994; van Echten et al, 1995). Variable amounts of loss have been revealed by allelotype analysis of various loci on 1p (Wang et al, 1980; Parrington et al, 1987; Mathew et al, 1994), including up to 45% LOH in one of these studies (Mathew et al, 1994). These results contrast with a recent CGH study of TGCT, which determined gain rather than loss of 1p material (Korn et al, 1996).

Loss of material at the subtelomeric region of 1p has been found as a common region of loss in various categories of germ cell tumours from young children. Cytogenetic analysis of GCT of the endodermal sinus type suggested a common area of deletion at 1p36 (Perlman et al, 1996). Interphase analysis using the marker for the PITSLRE locus at 1p36, a candidate tumour-suppressor gene for involvement in neuroblastoma, demonstrated loss of this locus in eight out of ten paediatric cases. Similarly, Stock et al (1994) demonstrated loss of the D1Z2 locus in four GCTs of children (two testicular embryonal carcinomas and two sacral yolk sac tumours). The present study shows that the D1Z2 locus is not commonly lost in the adult TGCT and larger regions of 1p are lost than in the paediatric cases. This difference may be significant in the different clinical and biological characteristics of adult and childhood GCTs.

Loss of material from chromosome 18 was found in over half of the cases studied. A high frequency of LOH has been reported for the *DCC* gene at 18q21.3, leading to the suggestion that loss of

© Cancer Research Campaign 1998 

function of this gene may be important in germ cell tumour pathogenesis (Murty et al, 1994). However, one cannot exclude the possibility that other genes might be significant. Loss of chromosome 5 was found in approximately one-third of cases and correlates with LOH reported at 5p15.1–p15.2, 5q11 and 5q34–q35 (Murty et al, 1996). Loss of chromosome 4 material, particularly 4p and 4q24–qter, was found in over half the cases and may be of significance. Similarly, specific loss of the 11q14–qter region was frequently found.

A number of common regions of gain were found as summarized in Table 2. These included gain of the 1p24–q31 region and the 2p13–pter region, including three cases with gain of 2p24–p25. Gain and amplification of this latter region has been noted and the *NMYC* gene suggested as a candidate (Korn et al, 1996). Gain of chromosome X, with a common overlapping region of Xq11–q21, was found. Klinefelter's syndrome (47, XXY) patients show an increased incidence of GCT and the cytogenetics of extra gonadal GCT and TGCT show an excess number of chromosome X (Mitelman, 1994). Taken together with our results, this suggests a role for the X chromosome, and possibly Xq11–q21 in particular, in the development of adult GCT.

In addition, some overlapping regions of copy number change were less frequent or not found in the seminoma compared with the non-seminoma. These included loss of 15q and chromosome 19 material and gain of 5q14–q23 and 6q21–q24. Loss of 13q was associated with SE whereas gain was noted in the NS and conversely for chromosome 22, loss was found in NS compared with gain in SE (Table 2). These results suggest regions that may contain genes involved in the divergent development of SE and NS. The reduced levels of expression of the RBI gene at 13q14 noted particularly in SE may reflect the chromosome dosage differences observed here (Strohmeyer et al, 1991). The difference in copy number of chromosome 15 and 22 material between SE and NS is consistent with previous cytogenetic and interphase FISH studies (Castedo et al, 1989*a*,*b*; Looijenga et al, 1993).

One SE and four NS were received after chemotherapy. The number and sites of copy number change in these did not appear to be significantly different from the other samples with the exception of gain of 6q21–q24, which warrants further investigation.

Determining significant rearrangements in the genome of TGCT amidst the considerable genomic instability is a difficult task. In contrast to molecular analysis of specific regions of the genome, the present study provides a global view of the copy number changes, and their frequency, which complements previous cytogenetic studies and indicates genomic regions for further study.

### ACKNOWLEDGEMENTS

The authors would like to thank the Cancer Research Campaign for supporting this study and the Association for International Cancer Research for providing a studentship for HG. The author also wishes to thank Martin Pera for providing the two cell lines, Richard Wooster for providing the PAC clones for chromosome 1, John Swansbury for assistance with the statistical analysis and Damian Smedley for his helpful comments.

### REFERENCES

Atkin N and Baker M (1983) A specific chromosomal marker in seminoma and malignant teratoma of the testis? *Cancer Genet Cytogenet* **10**: 199–204

Atkin N, Fox M, Baker M and Jackson Z (1993) Chromosome 12-containing markers, including two dicentrics, in three i(12p)-negative testicular germ cell tumours. *Genes Chrom Cancer* **6**: 218–221

Castedo SM, de Jong B, Oosterhius JW, Seruca R, te Meerman GJ, Dam A and Scraffordt Koops H (1989*a*) Cytogenetic analysis of ten human seminomas. *Cancer Res* **49**: 439–443

Castedo SM, de Jong B, Oosterhuis JW, Seruca R, Idenburg VJ, Dam A, te Meerman GJ, Scraffordt Koops H and Sleijfer DT (1989*b*) Chromosomal changes in primary testicular nonseminomatous germ cell tumours. *Cancer Res* **49**: 5696–5701

Damjanov I (1993) Pathogenesis of testicular germ cell tumours. *Eur Urol* **23**: 2–7

de Jong B, Oosterhuis JW, Castedo SMMJ, Vos AM and te Meerman GJ (1990) Pathogenesis of adult testicular germ cell tumours. A cytogenetic model. *Cancer Genet Cytogenet* **48**: 143–167

du Manoir S, Speicher MR, Joos S, Schrock E, Popp S, Dohner H, Kovacs G, Robert-Nicoud M, Lichter P and Cremer T (1993) Detection of complete and partial chromosome gains and losses by comparative genomic in-situ hybridisation. *Hum Genet* **90**: 590–610

Hemminki A, Tomlinson I, Markie D, Jarvinen H, Sistonen P, Bjorkqvist A-M, Knuutila S, Salovaara R, Bodmer W, Shibata D (1997) Localization of a susceptibility locus for Peutz-Jeghers syndrome to 19p using comparative genomic hybridisation and targeted linkage analysis. *Nature Genet* **15**: 87–90

Huddart RA, Wooster R, Horwich A and Cooper CS (1995) Microsatellite instability in human testicular germ cell tumours. *Br J Cancer* **72**: 642–645

Kallioniemi A, Kallioniemi O-P, Sudar D, Rutovitz D, Gray JW, Waldman F and Pinkel D (1992) Comparative genomic hybridisation for molecular cytogenetic analysis of solid tumours. *Science* **258**: 818–821

Korn W, Olde Weghuis D, Suijkerbuijk R, Schmidt U, Otto T, duManoir S, Geurts van Kessel A, Harstrick A, Seeber S and Becher R (1996) Detection of chromosomal DNA gains and losses in testicular germ cell tumours by comparative genomic hybridisation. *Genes Chrom Cancer* **17**: 78–87

Looijenga L, Gillis A, van Putten W and Oosterhuis J (1993) In situ numeric analysis of centromeric regions of chromosomes 1, 12, and 15 of seminomas, nonseminomatous germ cell tumours, and carcinoma in situ of human testis. *Lab Invest* **68**: 211–219

McManus AP, Gusterson BA, Pinkerton CR and Shipley JM (1995) Diagnosis of Ewing's sarcoma and related tumours by fluorescence in situ hybridisation detection of chromosome 22q12 translocations on tumour touch imprints. *J Pathol* **176**: 137–142

Mathew S, Murty VV, Bosl GJ and Chaganti RS (1994) Loss of heterozygosity identifies multiple sites of allelic deletions on chromosome 1 in male human germ cell tumours. *Cancer Res* **54**: 6265–6269

Mitelman F (1994) *Catalog of Chromosome Aberrations in Cancer*, 5th edn. Wiley-Liss: New York

Mostert M, van de Pol M, Olde Weghuis D, Suijkerbuijk R, Geurts van Kessel A, van Echten J, Oosterhuis J and Looijenga L (1996) Comparative genomic hybridisation of germ cell tumours of the adult testis: confirmation of karyotypic findings and identification of a 12p amplicon. *Cancer Genet Cytogenet* **89**: 146–152

Mostofi F and Sobin L (1977) International histological classification of testicular tumours (no. 6). In *International Histologic Classification of Tumours*. World Health Organization, Geneva.

Mukherjee A, Murty V, Rodriguez E, Reuter V, Bosl G and Chaganti R (1991) Detection and analysis of orgin of i(12p), a diagnostic marker of human male germ cell tumours, by fluorescence in situ hybridisation. *Genes Chrom Cancer* **3**: 300–307

Murty V, Li R, Houldsworth J, Bronson D, Reiter V, Bosl G and Chaganti R (1994) Frequent allelic deletions and loss of expression characterize the DCC gene in male germ cell tumours. *Oncogene* **9**: 3227–3330

Murty V, Reuter V, Bosl G and Chaganti R (1996) Deletion mapping identifies loss of heterozygosity at 5q15.1–15.2, 5q11 and 5q34–35 in human male germ cell tumours. *Oncogene* **12**: 2719–2723

Parrington JM, West LF and Povey S (1987) Loss of heterozygosity in hypertriploid cell cultures from testicular tumours. *Hum Genet* **77**: 269–276

Pera M, Blasco Lafita M and Mills J (1987) Cultured stem-cells from human testicular teratomas: The nature of human embryonal carcinoma, and its comparison with two types of yolk sac carcinoma. *Int J Cancer* **40**: 334–343

Perlman E, Valentine MB, Griffin CA and Look AT (1996) Deletion of 1p36 in childhood endodermal sinus tumours by two-color fluorescence in situ hybridisation: a pediatric oncology group study. *Genes Chrom Cancer* **16**: 15–20

Rodriguez E, Mathew S, Reuter VE, Ilson DH, Bosl GJ and Chaganti RS (1992) Cytogenetic analysis of 124 prospectively ascertained male germ cell tumours. *Cancer Res* **52**: 2285–2291

© Cancer Research Campaign 1998

Rodriguez E, Houldsworth J, Reuter VE, Meltzer P, Zhang J, Trent JM, Bosl GJ and Chaganti RSK (1993) Molecular cytogenetic analysis of i(12p) negative human male germ cell tumours. *Genes Chrom Cancer* **8**: 230–236

Samaniego F, Rodriguez F, Houldsworth J, Murty VV, Ladanyi M, Lele KP, Chen QG, Dmitrovsky E, Geller NL, Reuter V (1990) Cytogenetic and molecular analysis of human male germ cell tumours: chromosome 12 abnormalities and gene amplification. *Genes Chrom Cancer* **1**: 289–300

Sandberg AA, Meloni AM and Suijkerbuijk RF (1995) Reviews of chromosome studies in urological tumours. III. Cytogenetics and genes in testicular tumours. *J Urol* **155**: 1531–1556

Shipley J, Weber-Hall SJ and Birdsall S (1996) Loss of the chromosomal region 5q11-q31 in the myeloid cell line HL-60 is not associated with a 5q-chromosome; characterization by comparative genomic hybridisation and fluorescence in situ hybridisation. *Genes Chrom Cancer* **15**: 182–186

Skakkebaek N, Berthelsen J, Giwercman A and Muller J (1987) Carcinoma-in-situ of the testis: Possible origin from gonocytes, and precursor of all types of germ cell tumours except spermatocytoma. *Int J Androl* **10**: 19–28

Speicher M, Jauch A, Walt H, du Manoir S, Ried T, Jochum W, Sulser T and Cremer T (1995) Correlation of microscopic phenotype with genotype in a formalin-fixed, paraffin embedded testicular germ cell tumour with universal DNA amplification, comparative genomic hybridisation, and interphase cytogenetics. *Am J Pathol* **146**: 1332–1340

Stock C, Ambros IM, Lion T, Haas OA, Zoubek A, Gadner H and Ambros PF (1994) Detection of numerical and structural chromosome abnormalities in pediatric germ cell tumours by means of interphase cytogenetics. *Cancer* **11**: 40–50

Strohemeyer T, Reissmann P, Cordon-Cardo C, Hartmann M, Ackermann R and Slamon D (1991) Correlation between retinoblastoma gene expression and differentiation in human testicular tumours. *Proc Natl Acad Sci USA* **88**: 6662–6666

Suijkerbuijk R, Sinke R, Meloni A, Parrington J, van Echten J, de Jong B, Oosterhuis J, Sandberg A and Geurts van Kessel A (1993) Overrepresentation of chromosome 12p sequences and karyotypic evolution in i(12p)-negative testicular germ cell tumours revealed by fluorescence in situ hybridisation. *Cancer Genet Cytogenet* **70**: 85–93

Suijkerbuijk R, Sinke R, Olde Weghuis D, Roque L, Forus A, Stellink F, Siepman A, van de Kaa C, Soares J and Geurts van Kessel A (1994) Amplification of chromosome subregion 12p11.2-p12.1 in a metastasis of an i(12p)-negative seminoma: relationship to tumour progression? *Cancer Genet Cytogenet* **78**: 145–152

van Echten J, Oosterhuis JW, Looijenga LHJ, van de Pol M, Wiersema J, le Meerman GJ, Schafford Koops H, Sleijfer D.T. and de Jong B (1995) No recurrent structural abnormalities apart from i(12p) in primary germ cell tumours of the adult testis. *Genes Chrom Cancer* **14**: 133–144

Wang N, Trend B, Bronson DL and Fraley EE (1980) Nonrandom abnormalities in chromosome 1 in human testicular cancer. *Cancer Res* **40**: 796–802

Weber-Hall S, Anderson J, McManus A, Abe STN, Pinkerton R, Pritchard-Jones K and Shipley J (1996*a*) Gains, losses and amplification of genomic material in rhabdomyosarcoma analyzed by comparative genomic hybridisation. *Cancer Res* **56**: 3220–3224

Weber-Hall S, McManus A, Anderson J, Nojima T, Abe S, Pritchard-Jones K and Shipley J (1996*b*) Novel formation and amplification of the PAX7-FKHR fusion gene in a case of alveolar rhabdomyosarcoma. *Genes Chrom Cancer* **17**: 7–13

© Cancer Research Campaign 1998